# Critical Points and Traveling Wave in Locomotion: Experimental Evidence and Some Theoretical Considerations

**DOI:** 10.3389/fncir.2017.00098

**Published:** 2017-12-08

**Authors:** Philippe Saltiel, Andrea d’Avella, Matthew C. Tresch, Kuno Wyler, Emilio Bizzi

**Affiliations:** ^1^Department of Brain and Cognitive Sciences and McGovern Institute for Brain Research, Massachusetts Institute of Technology, Cambridge, MA, United States; ^2^Département de Neurosciences, Faculté de Médecine, Université de Montréal, Montréal, QC, Canada; ^3^Department of Biomedical and Dental Sciences and Morphofunctional Imaging, University of Messina, Messina, Italy; ^4^Laboratory of Neuromotor Physiology, Santa Lucia Foundation, Rome, Italy; ^5^Departments of Biomedical Engineering, Physical Medicine and Rehabilitation, and Physiology, Northwestern University, Chicago, IL, United States

**Keywords:** central pattern generator, locomotion, critical point shifts, traveling wave, temporal grid, hippocampus, Winfree’s phase singularities, spinal cord

## Abstract

The central pattern generator (CPG) architecture for rhythm generation remains partly elusive. We compare cat and frog locomotion results, where the component unrelated to pattern formation appears as a temporal grid, and traveling wave respectively. Frog spinal cord microstimulation with N-methyl-D-Aspartate (NMDA), a CPG activator, produced a limited set of force directions, sometimes tonic, but more often alternating between directions similar to the tonic forces. The tonic forces were topographically organized, and sites evoking rhythms with different force subsets were located close to the constituent tonic force regions. Thus CPGs consist of topographically organized modules. Modularity was also identified as a limited set of muscle synergies whose combinations reconstructed the EMGs. The cat CPG was investigated using proprioceptive inputs during fictive locomotion. Critical points identified both as abrupt transitions in the effect of phasic perturbations, and burst shape transitions, had biomechanical correlates in intact locomotion. During tonic proprioceptive perturbations, discrete shifts between these critical points explained the burst durations changes, and amplitude changes occurred at one of these points. Besides confirming CPG modularity, these results suggest a fixed temporal grid of anchoring points, to shift modules onsets and offsets. Frog locomotion, reconstructed with the NMDA synergies, showed a partially overlapping synergy activation sequence. Using the early synergy output evoked by NMDA at different spinal sites, revealed a rostrocaudal topographic organization, where each synergy is preferentially evoked from a few, albeit overlapping, cord regions. Comparing the locomotor synergy sequence with this topography suggests that a rostrocaudal traveling wave would activate the synergies in the proper sequence for locomotion. This output was reproduced in a two-layer model using this topography and a traveling wave. Together our results suggest two CPG components: modules, i.e., synergies; and temporal patterning, seen as a temporal grid in the cat, and a traveling wave in the frog. Animal and limb navigation have similarities. Research relating grid cells to the theta rhythm and on segmentation during navigation may relate to our temporal grid and traveling wave results. Winfree’s mathematical work, combining critical phases and a traveling wave, also appears important. We conclude suggesting tracing, and imaging experiments to investigate our CPG model.

## Introduction

This review article will primarily be an account of how, in line with this Frontiers topic, work in a lower vertebrate (frog) spinal cord added insight to previous work investigating the central pattern generator (CPG) for cat locomotion, and how both approaches enrich each other. Although the focus of this review will be influenced by the fact that one of the authors was involved in research in both species, other relevant work will also be reviewed.

We will begin by presenting evidence that the CPG for cat locomotion conceptually consists of two components. We will then present how research in the frog led to insights into each of these two components. We will also review how broader perspectives coming from the hippocampal and medial entorhinal circuitry on one hand, and from mathematical work on phase singularities on the other hand, might further inform insights derived from comparing the results in cat and frog locomotion. We will conclude with a suggestion for future directions.

## Results for Cat Locomotion

**Figure 1:**
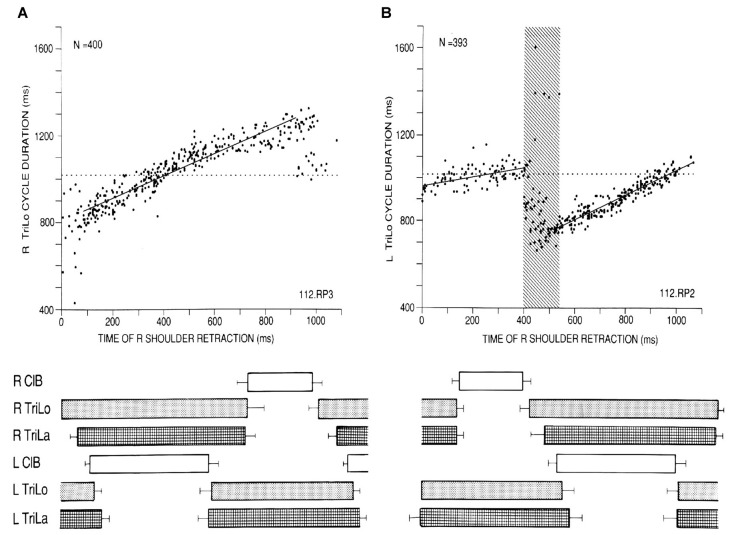
Critical point in the cat locomotor cycle identified with phasic R shoulder retractions. **(A)** Effect on R TriLo cycle duration, according to the time of retraction measured from R TriLo onset. **(B)** Effect on L TriLo cycle duration, with retraction time measured from L TriLo onset. Boxes below the graphs show the unperturbed cycle structure, and help to visualize when the perturbation was applied. Together these two graphs identified a critical point centered at the transition from R ClB to R TriLo, which corresponds in real locomotion to the transition between swing and stance. This corresponds to critical point D in Figure 3. ClB, Cleidobrachialis (shoulder protractor and elbow flexor); TriLo, long head of Triceps (shoulder retractor and elbow flexor); TriLa, lateral head of triceps (pure elbow extensor). Dotted lines indicate control cycle duration. Reproduced with permission from Saltiel and Rossignol (2004a).

**Figure 2:**
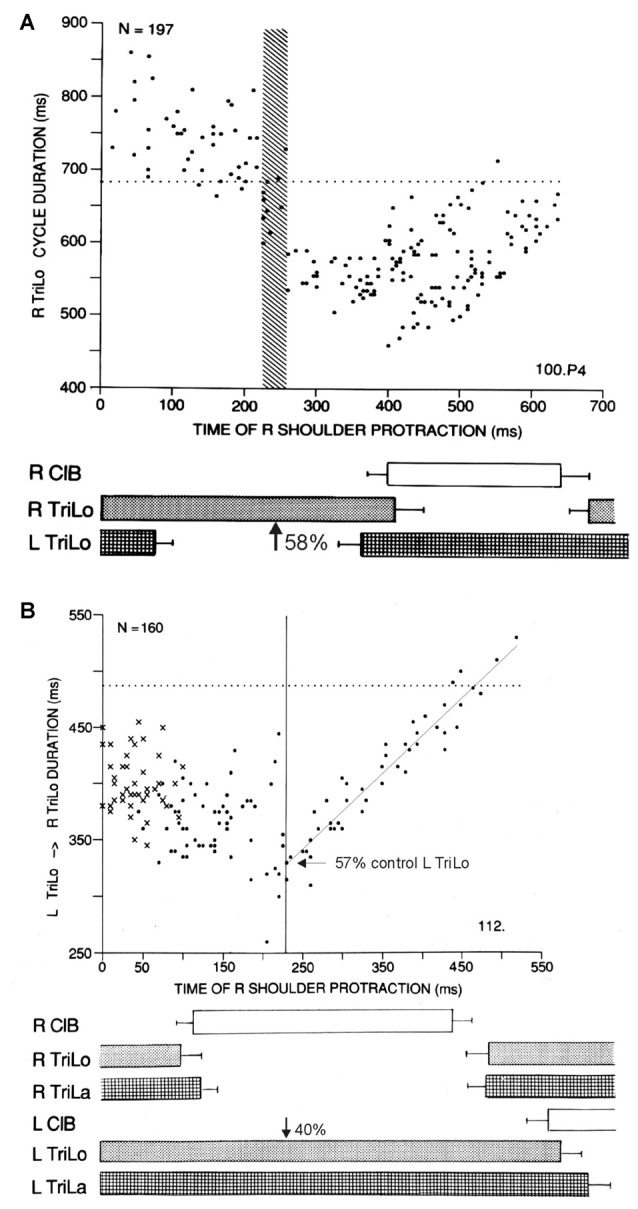
Two other critical points in the cat locomotor cycle, identified with phasic R shoulder protractions. **(A)** Effect on R TriLo cycle duration, according to the time of protraction measured from R TriLo onset. There is a critical point at ~58% of R TriLo burst, where the effect on the cycle abruptly changes from lengthening to shortening. The dotted line indicates control cycle duration. This critical point is labeled C in Figure 3. **(B)** Effect on the L TriLo onset to R TriLo onset interval (essentially L stance onset to R stance onset). The dotted line indicates the control duration of that interval. There is a critical point at 40% of L TriLo burst, past which the advance in R TriLo onset by R shoulder protractions follows a linear relationship. This critical point is labeled B in Figure 3. It is also noted on the ordinate, that at this critical point B, the R TriLo onset is advanced to ~57% of the control L TriLo burst, that is point D is advanced to point C (more precisely to the symmetrical point C, half-a-cycle away from the point C identified in **A**). Reproduced with permission from Saltiel and Rossignol (2004a).

We begin with some insights into the CPG for cat locomotion, derived from a “black box” approach, using the effects of phasic and tonic proprioceptive inputs on the fictive locomotor output recorded from both forelimbs (Saltiel and Rossignol, 2004a,b). In the fictive preparation, there is no movement as the muscles are paralyzed with curare, and the CPG activity is recorded from muscle nerves (electroneurograms = ENGs). When phasic shoulder perturbations are applied to one forelimb at various times in the locomotor cycle, measured from the onset of the long head of triceps (TriLo, shoulder retractor and elbow extensor), abrupt transitions are seen in their effects, identifying critical points. Retractions monotonically shorten, then lengthen cycle duration (interval between consecutive TriLo onsets) compared to control, but at the flexor to extensor transition (labeled critical point D), they produce a bifurcation where the cycle is either shortened back to control duration, or remains lengthened (Figure 1A). The upper arm of the bifurcation in Figure 1B corresponds to the activation of a second flexor burst, while the contralateral extensor remains active (2:1 rhythm). Protractions during the extensor cycle at first prolong it, but at ~55% of the extensor phase (labeled critical point C), this effect abruptly changes to a shortening (Figure 2A). Conceptually, the plots of Figures 1, 2A are similar to studying phase response curves (Winfree, 1977). The methodological difference is that we plot the perturbed cycle duration against the time of the perturbation, whereas phase response curves plot the phase shift resulting from the change in cycle duration (positive/negative shift, or phase advance/delay, for a shortened/prolonged cycle), against the phase of the perturbation.

Protractions during the flexor phase shorten it, but at ~40% of the contralateral extensor phase (labeled critical point B), an abrupt transition occurs where protraction maximally advances the onset of the ipsilateral extensor to ~55% of the contralateral extensor phase, followed by a linear relationship (Figure 2B). Thus these phasic perturbations identified three critical points in the locomotor cycle. An interplay between these critical points is already seen, since the last result can be reformulated as saying that the effect phasic protractions at point B, is to advance point D to point C.

We found several other lines of evidence strengthening the identification of these critical points.

First, the effects of phasic retractions and protractions on the ipsilateral extensor cycle duration are markedly similar respectively to those produced by a brief hyperpolarizing or depolarizing stimulus on the inter-spike interval of a cyclically firing neuron (Winfree, 1987, his Figures 4.3 and 4.1). Like the effect of retraction on the cycle, a brief hyperpolarizing pulse first shortened, then lengthened the inter-spike interval, and near the end of the interval, produced a phase singularity in the work of Best and Winfree (Best, 1979). And like protraction, a brief depolarization first lengthened, then shortened the inter-spike interval, with a phase singularity in between. Thus it appears that a very basic result found in a single firing neuron is conserved at the level of an entire CPG. A recent study of human locomotion (Funato et al., 2016), using a novel method to obtain phase response curves to treadmill acceleration and deceleration, found the same two key points in the cycle as we did: one at touchdown (like our point D), and one at mid-stance (like our point C). A difference was that these points were not described as phase singularities, but as phases to which the cycle was preferentially reset (no change in phase when perturbation was done at that phase, phase advance and delay for perturbations prior to and after that phase respectively).

**Figure 3:**
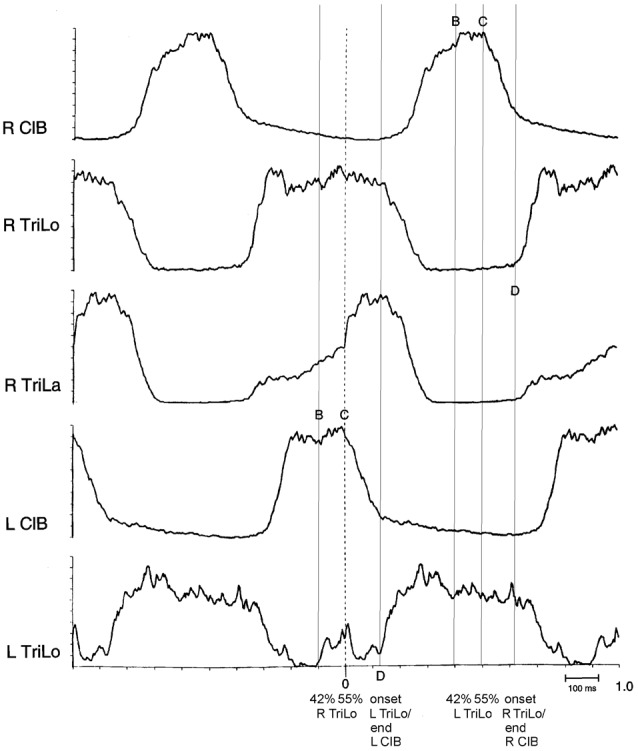
Structure of the fictive cat locomotor cycle, and location of critical points. Note the different burst shape of TriLo and TriLa, with an inflection point in TriLa, on which this average is synchronized. The location of critical points B, C and D identified in Figures 1, 2 is indicated in both half-cycles by vertical lines. Critical point B is at ~40% of TriLo burst. Critical point C is at ~55% of TriLo burst, and simultaneously coincides with the inflection in TriLa, and the onset of descent in the contralateral ClB. Critical point D is at TriLo onset/end of ClB. Reproduced with permission from Saltiel and Rossignol (2004a).

Second, some of the critical points correspond to clear-cut events in the morphology of the ENG bursts (Figure 3). Point D essentially corresponds to the transition from the ipsilateral flexor to the ipsilateral extensor phase. Point C corresponds to an abrupt upward inflection in the ipsilateral lateral head of triceps (TriLa, elbow extensor), and the onset of descent of the contralateral cleidobrachialis (ClB, shoulder protractor, and elbow flexor). This correspondence of critical points with ENG burst events further suggests that they are built in the CPG program, and that at least point C assists in interlimb coordination.

Third, the timing of these critical points in the fictive locomotor cycle closely correlates with important biomechanical events in the real locomotor cycle (Manter, 1938). For example, point C corresponds to an upward inflection in the ipsilateral vertical ground reaction force (GRF), a reversal from backwards to forwards in the ipsilateral horizontal GRF, and onset of contralateral elbow extension during swing (transition from F to E1 kinematic phase). Point B corresponds to when, after the initial shoulder retraction and elbow flexion necessary in the early swing phase to clear the ground, the limb movement changes to protraction. Thus not only are the critical points embedded in the CPG program, but they are at times that are biomechanically meaningful.

The most interesting result came from examining the effect of tonic proprioceptive perturbations on the structure of the fictive locomotor cycle (Saltiel and Rossignol, 2004b). First, we found that the interlimb coupling at point C (ipsilateral Trila inflection, contralateral ClB onset of descent) remained preserved during tonic shoulder protractions and retractions, and tonic elbow flexions and extensions. Second, when we synchronized on this preserved coupling to compare the control (limb pendent), and perturbed cycles (limb in a different tonic position), we observed that the reorganization in terms of burst duration was again based on the critical points. For example, tonic shoulder protraction/retraction prolonged/shortened ipsilateral TriLo and contralateral ClB only in their portions before point C. Overall the timing of burst events (e.g., offset, onset) remained unchanged in approximately the half-cycle from point C to the following point B with shoulder perturbations. Tonic elbow flexion instead prolonged the portion of the extensor burst after point C.

**Figure 4:**
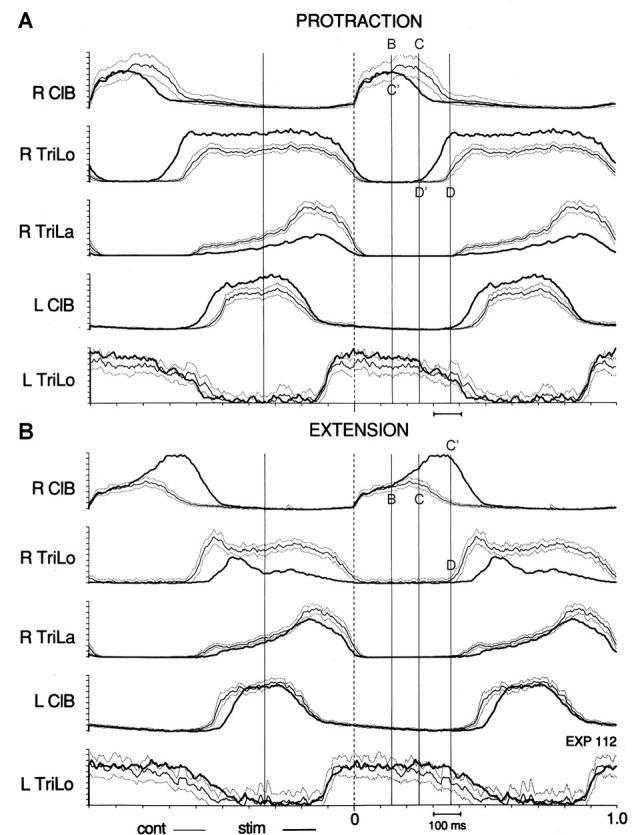
Effect of tonic protraction and tonic extension: shifts in critical points. Averages of the control (thin line, with dotted lines one standard deviation (SD) away) and perturbed (thick, darker line) cycles are synchronized on R ClB onset. Vertical lines are drawn at the same times in **(A,B)**, corresponding to the critical points in the control cycle shown in Figure 3. **(A)** Tonic protraction shortens R ClB and prolongs R TriLo. R TriLo onset is shifted from D to D’, but D’ corresponds to the time of point C in the control cycle. The onset of descent of R ClB is shifted from C to C’, but C’ corresponds to the time of point B in the control cycle. **(B)** Tonic extension increases R ClB amplitude, starting at point B, and prolongs R ClB. The onset of descent of R ClB is shifted from C to C’, but C’ corresponds to the time of point D in the control cycle. These shifts between critical points suggest that a temporal “grid” is preserved during the changes in burst durations produced by the tonic perturbations. Reproduced with permission from Saltiel and Rossignol (2004b).

More specifically, we observed that shifts between critical points accounted for the changes in burst durations. For example, during tonic protraction, the onset of descent of ClB (point C) is advanced to the timing of point B in the control cycle; and similarly the onset of Trilo (point D) is advanced to the timing of point C in the control cycle. Thus we can summarize this by saying that tonic protraction changes burst durations by producing shifts between critical points: point C is shifted to C’ = B ; point D is shifted to D’ = C (Figure 4A). During tonic extensions and tonic retractions, ipsilateral ClB is prolonged; this happens by delaying the onset of descent of ClB (point C) to the timing of point D in the control cycle, i.e., point C is shifted to C’ = D (Figure 4B). Changes in burst amplitudes seem generally not to be restricted to segments delimited by the critical points. A notable exception is during tonic elbow extension, where the ipsilateral ClB remains of unchanged amplitude initially, and then increases at point B (Figure 4B).

These results suggest that when burst durations are changed by tonic proprioceptive inputs, something important actually remains unchanged, i.e., a temporal structure or grid, which allows the shift of a critical point to the timing of the earlier or later critical point in the sequence.

In other words, the cat locomotor CPG appears to be conceptually made up of two components, ENG burst segments delimited by critical points; and a temporal structure or grid determining where these critical points may be located in time.

Other researchers have suggested two components to the CPG (Lennard, 1985; Burke et al., 2001; Rybak et al., 2006). This is based on a different type of evidence, i.e., observations that spontaneous EMG deletions (Stein and Daniels-McQueen, 2004), or the effects of afferent stimulation, may or may not be associated with permanent resetting of the rhythm. Although formulations of rhythm-generation and pattern-formation in CPG models (Perret and Cabelguen, 1976; Burke et al., 2001; Lafreniere-Roula and McCrea, 2005; McCrea and Rybak, 2008; Zhong et al., 2012) do not involve critical points, they postulate, like our results, a network concerned with temporal organization, that constrains the expression of EMG patterns.

From a broader perspective, it is interesting that Cordo et al. (1993) found, when comparing human reaching and grasping to different heights, or with different hand orientations, that EMG averages only diverged at discrete periodic times in the movement. They concluded that a central timing oscillator seemed involved even in a non-cyclical movement sequence. More recently, Lisman (2014) reviewed evidence for discontinuous control of the motor system, in particular for action selection, by ~10 Hz oscillations.

## Results in The Frog

Around the same time that this work (Saltiel and Rossignol, 2004a,b) was being done in the cat, a new approach was being developed at MIT. Inspired by the equilibrium-point hypothesis (Feldman, 1986; Bizzi et al., 1992), the question was whether circuits exist in the spinal cord, that specify limb movement in particular direction, to mediate commands from higher brain centers. Instead of a black-box approach, the method used was a direct electrical micro-stimulation of the intermediate region of the lumbar spinal cord in the spinalized frog. A modular organization was found, where only a limited set of force directions could be produced, with intermediate force directions obtained by co-stimulation of spinal cord sites (Bizzi et al., 1991).

In order to avoid stimulation of fibers of passage, chemical micro-stimulation was next done, using focal intra-spinal iontophoresis (Saltiel et al., 1998). The chemical that was found to work was N-methyl-D-Aspartate (NMDA). Only 24% of tested sites respond to NMDA, and with the parameters used, the spread of NMDA by the average time that forces began was estimated at 150–270 μm radius, which corresponds to less than 1% of the volume of a spinal cord segment.

With NMDA, a minority of the active spinal cord sites responded with a single tonic force, which could be rostral flexion, adduction, caudal extension, or lateral extension. The more common effect was to obtain a rhythm made up of an alternation between these forces, with some preferred combinations of these forces being more frequently observed. The tonic forces were topographically organized, and interestingly, the spinal cord rhythms were obtained from sites near the tonic regions producing their constituent forces, as well as from a common caudal region in the cord. It is not surprising that NMDA often produced a rhythm, since it is a very common agent used to activate CPGs (Wallén and Grillner, 1987; Li, 2011). The above results provide strong evidence that the modules identified in the frog spinal cord could represent building blocks of CPGs. The hypothesis of modularity of CPGs had already been formulated by other investigators (Grillner, 1981; Jordan, 1991).

Thus these modules identified in the frog could be related conceptually to one of the CPG components suggested for the cat, i.e., the production of units of muscle activations, that here would bring the limb in specific directions.

**Figure 5:**
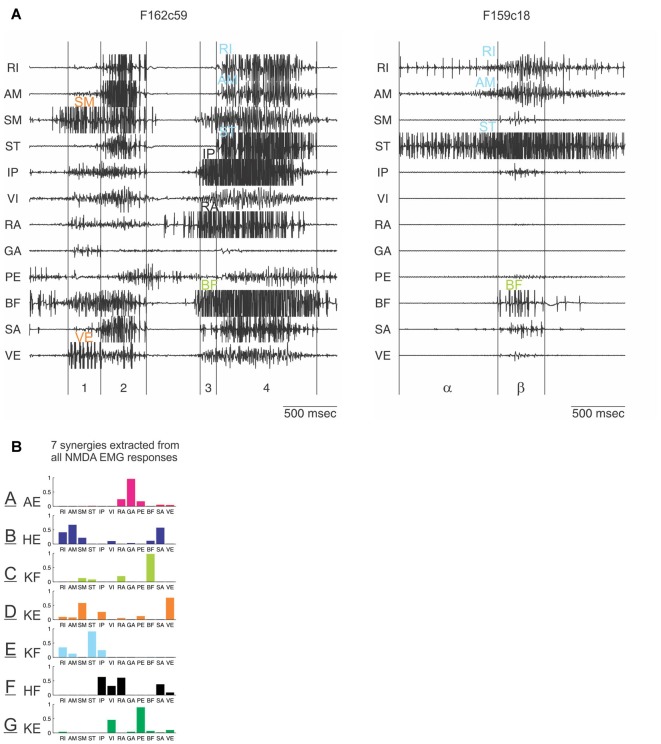
Identification of synergies entering in the composition of EMG patterns elicited by N-methyl-D-Aspartate (NMDA) iontophoresis in the frog spinal cord. **(A)** Examples of EMG patterns from 12 recorded muscles in the hindlimb. Visually comparing many such patterns, and how they evolve in time, led to the distinct impression that they are made of smaller subunits, indicated with different colors. **(B)** This was formally demonstrated with a computational algorithm which extracted a set of seven muscle synergies whose combinations reconstructed the EMG patterns. These synergies are labeled A to G (color code similar to the one used in **A**). Their main biochemical action is indicated: AE, ankle extensor; HE, hip extensor; KF, knee flexor; KE, knee extensor; HF, hip flexor. Muscle abbreviations: RI, rectus internus; AM, adductor magnus; SM, semimembranosus; ST, semitendinosus; IP, iliopsoas; VI, vastus internus; RA, rectus anterior; GA, gastrocnemius; PE, peroneus; BF, biceps femoris; SA, Sartorius; VE, vastus externus. Reproduced with permission from Saltiel et al. (2001, 2016).

To explore this idea further, the next step was to analyze the EMG recordings produced by NMDA in the frog spinal cord. Looking carefully at EMG patterns, it was realized that these appear composed of smaller units which combine to produce the full pattern (Saltiel et al., 2001). For example, an EMG pattern could sometimes be seen to evolve over time, beginning with a subset of EMGs, to which another subset of EMGs was later added (Figure 5A). In other circumstances, the subsets of EMGs were produced in isolation. These smaller units were termed muscle synergies. EMG patterns evoked in natural behaviors also appeared to represent a combination of synergies (Tresch et al., 1999; d’Avella et al., 2003; Ting and Macpherson, 2004; Cheung et al., 2005).

In order to formally extract these muscle synergies suspected from visual inspection, a computational algorithm was developed (Tresch et al., 1999). Starting from a random set of initial synergies, the algorithm finds the coefficients of activation of synergies whose combinations best reconstruct the data. In the subsequent iterations, the algorithm alternatively updates the synergies, and the coefficients to maximize the EMG data reconstruction; typically a non-negative least-squares method is used. It was found that the combination of seven muscle synergies, labeled as A to G (Figure 5B), explains 91% of the variance of the NMDA-evoked EMGs recorded from 12 muscles (Saltiel et al., 2001).

It is possible to extend the above observations about NMDA evoking rhythms suggestive of CPG activation, by examining the EMG patterns. Thus we found that one of the commonly evoked rhythms, adduction-caudal extension-flexion to body, consists of 2 rhythm subtypes determined on the basis of EMGs (Saltiel et al., 2005). Subtype 1 consists of an adduction based on synergy E, and caudal extension based on synergies A + B; in subtype 2, the adduction is based on synergies E + C, and caudal extension on synergies A + D. Flexion onsets also differ between these 2 subtypes. Topographically, these 2 rhythms appear to originate from interleaved regions in the spinal cord encoding in rostrocaudal order, CE2, ADD1, ADD2 and CE1 (where CE = caudal extension, ADD = adduction and 1 and 2 refer to the two subtypes of linked caudal extensions and adductions). CE1 and CE2 caudal extensions appear to relate respectively to jumping and swimming extensions (Saltiel et al., 2005).

**Figure 6:**
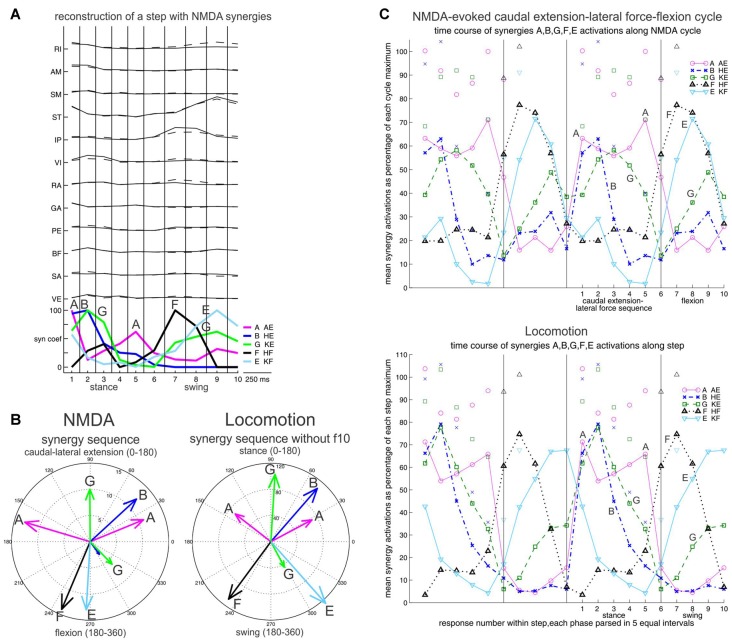
Synergy sequence in frog locomotion, and comparison with the NMDA-evoked caudal extension-lateral force-flexion cycle. Stance and swing, or caudal extension-lateral force and flexion are each divided in five equal bins. **(A)** Reconstruction of a step with NMDA synergies, illustrating the A-B-G-A-F-E-G synergy sequence. **(B)** The mean times of synergy activation peaks are shown in angular histograms for the NMDA and the locomotor cycle. The difference between B and G during stance was a bit less striking when including frog f10, but remained strongly significant. **(C)** Time course of synergy activations shown in averages. Again a similar A-B-G-A-F-E-G synergy sequence is seen in the NMDA caudal extension-lateral force-flexion cycle, and locomotion. Averages are shown twice side-by-side to better visualize the phase transitions. Symbols above traces represent one SD. Reproduced with permission from Saltiel et al. (2016).

What about locomotion? Frogs are also capable of terrestrial quadrupedal walking (Roh et al., 2011). Here we had the intuition that NMDA-evoked rhythms comprising a lateral extension force, and in particular a commonly observed CE1-lateral force sequence would be the most relevant to locomotion. We reconstructed the locomotor EMG data with the NMDA synergies, and found close similarity between the locomotor synergy sequence, and the synergy sequence of the NMDA CE1-lateral force-flexion cycle (flexion onset without the C synergy). In both cases, an A-B-G-A-F-E-G synergy sequence is seen (Figure 6; Saltiel et al., 2016). Of note, the synergies are not activated in complete isolation in this sequence, but partly overlap; for example, during stance, synergies B and G begin together at the same time as the first synergy A peak, but synergy G outlasts them.

**Figure 7:**
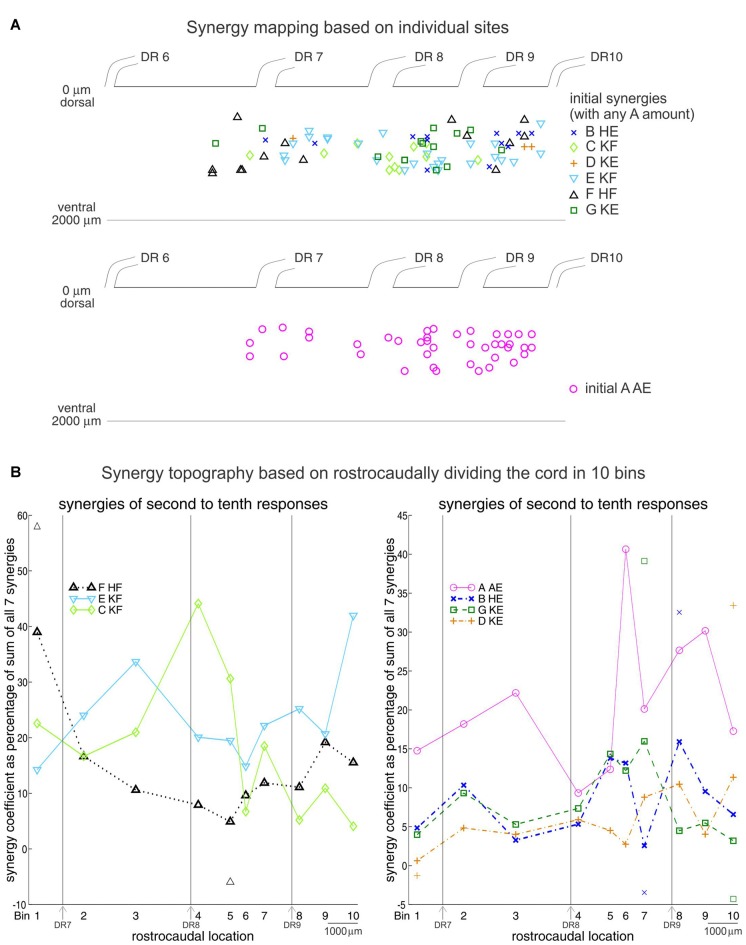
Synergy topography in the frog spinal cord. **(A)** Location of the spinal cord sites encoding individual synergies. Upper panel: a site was considered to encode synergy B, C, D, E, F, or G when activation of that synergy in the initial responses exceeded each of the other five synergies by a ratio ≥1.733 (arctangent ≤ 30°). Any amount of synergy A was allowed. Lower panel: a site encoded synergy A when its activity in the initial responses exceeded each of the other six synergies by a ratio ≥1.733. **(B)** Rostrocaudal topography of synergies A-G based on the second to tenth set of responses in the NMDA-evoked output. One-hundred and ten sites were divided rostrocaudally in 10 bins. Numbers identify the bin centers, and the 7th, 8th and 9th dorsal roots locations are indicated. For each bin, the percentages that synergies A–G contributed to the second to tenth set of responses at each site were pooled together, averaged and plotted. Symbols above and below traces represent one SD. SDs were 35.5 and 17.5% at bins 6 and 4 for synergy A, 32 and 7.3% at bins 4 and 10 for synergy C, and 35.2 and 13.8% at bins 10 and 1 for synergy E. Reproduced with permission from Saltiel et al. (2016).

To understand how this sequence might come about, we then turned to the topography of NMDA synergies in the lumbar cord (Saltiel et al., 2016). This was determined by looking at the early EMG responses evoked at 110 spinal cord sites. Although there is considerable overlap, each of the seven synergies is preferentially activated from distinct regions of the spinal cord (Figure 7A), as seen in a pattern of peaks and troughs of activation along the rostrocaudal axis of the cord (Figure 7B). Other researchers using optical stimulation (Hägglund et al., 2013; Levine et al., 2014), or recordings (Tresch and Kiehn, 1999), have also found evidence for a rostrocaudal topography of motor patterns, although generally classified as flexor or extensor, rather than precisely identified synergies.

**Figure 8:**
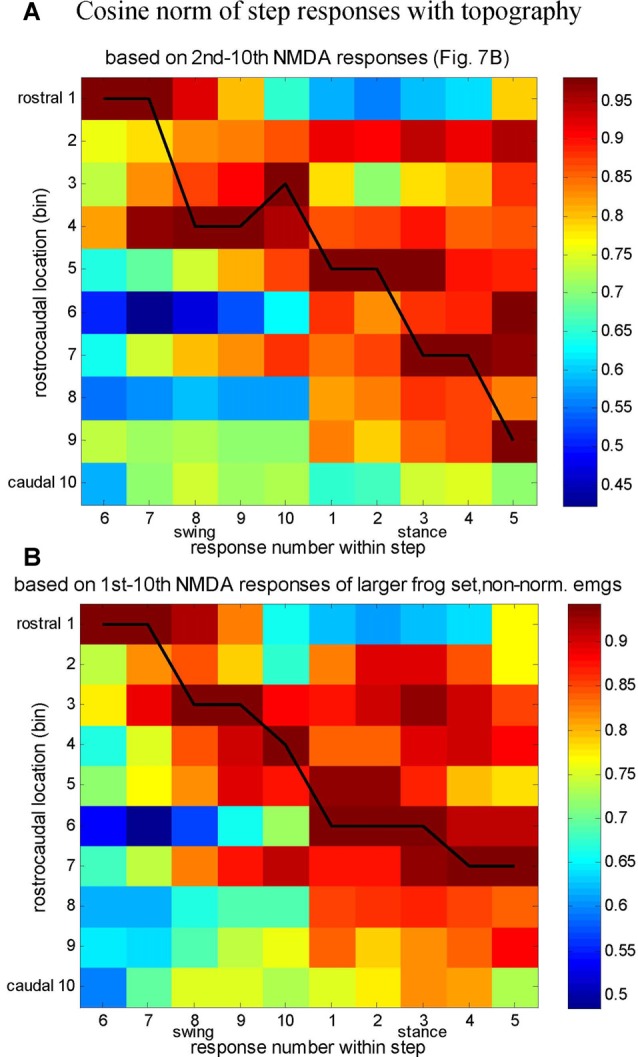
Comparison between the synergy composition of successive responses in the locomotor cycle, and synergy rostrocaudal topography. **(A)** Cosine angle between the average synergy composition of step responses (6–10, swing and 1–5, stance), and the 10 bins synergy topography based on the second to tenth NMDA responses (Figure 7B). The normalized dot products between the seven-synergy vector of each step response and the seven-synergy vectors for each rostrocaudal bin are plotted in pseudocolor. The black line joins the highest matches between the step responses and the synergy topography (highest cosine angle). **(B)** Similar analysis, but with the topography based on non-normalized NMDA EMGs (same as for locomotion), the first to tenth NMDA responses, and an additional frog set (total of 168 sites). Both plots suggest a rostrocaudal progression of activity along the step cycle. Reproduced with permission from Saltiel et al. (2016).

Interestingly, the temporal sequence order of synergies in locomotion, and the rostrocaudal spatial sequence order of synergies in the spinal cord clearly show similarities (compare Figures 6C, 7B), and this was quantitatively demonstrated as well (Figure 8; Saltiel et al., 2016). Therefore it appears that the synergy topography is such that a rostrocaudal traveling wave of activation would activate them in the proper sequence for locomotion. In our article, this was also investigated as a model, using as in the literature, a pattern-formation layer (PF), here encoding muscle synergies; and a “traveling wave” layer (TW), giving some perpendicular external inputs to the PF layer, in particular to nodes producing synergy A. This model reproduced the locomotor synergy sequence, and allowed to explore possible modifications by afferent inputs. It should be noted that the connectivity between synergy nodes within the PF layer in this model is already sufficient to give a propagating wave of synergy activation. However the external inputs from the TW layer to the PF layer are necessary to obtain the double-peaked synergy A activation during stance, contribute to determining stance onset, and participate in modifications by afferent inputs.

After finding this evidence for a traveling wave in frog locomotion, we noticed that a traveling wave at the interneuronal level had already been reported in the fictive scratch cycle of the cat (Cuellar et al., 2009), as shown by recording of cord dorsum potentials, even after ventral horn removal; and by interneuronal recordings. Prior to this, among limbed vertebrates, traveling waves of motoneuron activation had been reported in the newt (Delvolvé et al., 1997), and the rodent (Bonnot et al., 2002; Cazalets, 2005), and suggested in the cat (Yakovenko et al., 2002), and human (Ivanenko et al., 2006). In vertebrates without limbs, a traveling wave occurs during swimming in the lamprey (Cohen et al., 1992), and zebrafish (Wiggin et al., 2012). Among invertebrates with limbs, a traveling wave occurs in the crustacean swimmeret system (Davis, 1969; Heitler, 1980). More recently, a traveling wave of interneuronal activation has been reported by examining oscillator ensembles in the Aplysia pedal ganglion during locomotion (Bruno et al., 2015). These interneuronal ensembles both show rotational dynamics as seen in a principal component analysis, and physical rotation of activity, demonstrated as an elliptical trajectory when physically mapped on the ganglion. It has also been shown that a two-dimensional spinal cord model could generate traveling waves accounting for the various quadruped gaits (Kaske et al., 2003).

What could the neural substrate be for a rostrocaudal traveling wave? The first idea would be a descending excitatory pathway, since it is generally accepted that an excitatory mechanism is at the core of the locomotor CPG (Grillner and El Manira, 2015). In the last 10–15 years, genetic methods have allowed to identify locomotor functions for several of the V0 to V3 interneurons defined by their transcription factors (Kiehn, 2016), and to suggest what their connectivity might be (Rybak et al., 2015). Among these, the excitatory V2a interneurons, which exist in the mouse, zebrafish, *Xenopus* tadpole and lamprey, have several features that might make them good candidates to participate in a rostrocaudal wave of excitation within the PF layer. First, V2a interneurons of type II in the mouse, which become silent during non-resetting deletions, are believed to be part of the PF layer and to recruit motoneurons (Zhong et al., 2012; Rybak et al., 2015). Second, V2a interneurons appear from optogenetic experiments to also be connected with other V2a interneurons, and their selective activation is sufficient to produce the rostrocaudal wave of swimming in the zebrafish (Ljunggren et al., 2014). Third, their axons are ipsilaterally descending, at least in the zebrafish (Kimura et al., 2006; Ljunggren et al., 2014). One may thus ask whether V2a type II interneurons might be premotor interneurons projecting both to motoneurons to encode synergies, and to other premotor neurons further caudally, to participate in a rostrocaudal wave of excitation producing the locomotor synergy sequence as in our model (Saltiel et al., 2016).

In the mammalian CPG literature, genetic studies of intralimb coordination have focused on flexor-extensor alternation, probably for two reasons. First, the L2 and L5 ventral roots show alternating activity during *in vitro* neonatal rat locomotor activity, with L2 in phase with simple flexors, and L5 with extensors, although the EMG pattern is in fact more complex (Kiehn and Kjaerulff, 1996). Second, whereas a longitudinally traveling wave is a well-established concept for limbless vertebrates such as the lamprey or zebrafish, it is only more recently that it has been proposed as a mechanism for locomotion of vertebrates with limbs (see above). Nevertheless, it is interesting that the V1 and V2b inhibitory interneurons, which are key for flexor-extensor alternation in the mouse, are respectively homologous to an ascending inhibitory interneuron (aIN in *Xenopus* tadpole, and CiA in zebrafish), and to a descending inhibitory interneuron (VeLD; Zhang et al., 2014). Further, V1-derived (ascending) inhibition promoted limb extension, and V2b-derived (descending) inhibition promoted limb flexion in the mouse (Britz et al., 2015). Given the greater rostral representation of flexor synergies, and generally greater caudal representation of extensor synergies in the frog spinal cord (Saltiel et al., 2016), these are the results we would expect if similar longitudinal inhibitory pathways also operate in the adult frog. The advantage of the postulated rostrocaudal traveling wave is that it does not reduce the activation of the seven synergies in the frog locomotor sequence, or the complex EMG patterns in other species, to just flexor-extensor alternation. At the same time, mechanisms for flexor-extensor alternation, and for a traveling wave are not incompatible. In particular, the ascending inhibitory pathway is active during swimming in *Xenopus* tadpole, and may be a candidate for regulating intersegmental delay (Li et al., 2004; Zhang et al., 2014). The combination of descending excitation, as discussed above (see also Cowley et al., 2010), and ascending inhibition, would be expected to favor a rostrocaudally traveling wave, as pointed out by Kimura et al. (2006). Such combined descending excitation and ascending inhibition between the more rostral flexor, and more caudal extensor synergies, could also relate to the observations of tighter phase-locking at the extension to flexion (E to F) than at the F to E transition (Boothe et al., 2013). These authors suggested a tighter phase-locking would be expected for a transition resulting from an escape or release from inhibition (here postulated from E to F).

In summary, examination of the genetic literature on CPG interneuronal components in different species, suggests hypotheses about how a traveling wave mechanism for locomotion in the limbed vertebrate might be implemented.

There is evidence that traveling waves may relate to the rhythm-generator (RG) component in the two-layer model of the CPG, and thus be involved in time-keeping. In resetting deletions of the motor output of fictive scratch in the cat, where the cycle period is not maintained, the cord dorsum potential rostrocaudally traveling wave simultaneously disappears. By contrast, the wave is maintained during non-resetting deletions. Both observations have been reproduced in a longitudinal two-layer CPG model with a traveling wave (Pérez et al., 2009).

When thinking back about our results in cat locomotion, we had already related the synergy modules in the frog, to the segments of ENG burst activation in the cat. Now with this new result of a traveling wave in frog locomotion, it seems natural to ask whether it could relate in some way to our other result in cat locomotion, i.e., the temporal grid or structure.

It is not straightforward to answer this question. However we would like to suggest that perhaps one way to approach it is to think about parallels with the literature on the hippocampus/medial entorhinal cortex (mEC) circuitry.

## Possible Insights from Some Similarities to Hippocampus/Entorhinal Circuitry

One may see elements of similarity between spatial navigation of a locomoting animal, controlled by the hippocampus and mEC, and the navigation of the limbs in space during locomotion, under control of the CPG. One may speak of limb navigation during the swing phase for example because there are constraints such as clearing the ground at take-off, or coordinating different joints during limb protraction, and then during landing. In navigation of the whole animal, place cell activity is determined by both self-motion and environmental inputs. During locomotion, the limb EMGs are both determined by a central program and by afferent input.

This idea of similarity is also supported by comparing what happens to place cell activity when a rat linear trajectory is shortened by putting the start-box closer to the goal location, and what happens in cat fictive locomotion during tonic forelimb protraction which shortens the swing phase. In the former case, one may see compared to the control trajectory, an abrupt shift from place cells active near the start-box to place cells active farther along the trajectory, with disappearance of place cells for intermediate locations, even though these locations are still being traversed (Gothard et al., 1996, their Figure 8). In the latter case, one sees a disappearance of the intermediate segment of ClB (shoulder protractor and elbow flexor) electroneurogram (segment B-C in Figure 4A), corresponding to an abrupt shift directly of critical point C to B, when comparing to the control locomotor output (Saltiel and Rossignol, 2004b).

We already mentioned that we could approximately relate our results in frog and cat locomotion by considering synergy modules in the frog equivalent to the ENG burst segments in the cat. This suggests that the other component for locomotor generation in the frog, i.e., a traveling wave, might be equivalent to the other locomotor component in the cat, i.e., a temporal grid. Perhaps the analogy with the hippocampus and mEC can give glimpses into how a traveling wave could relate to a temporal grid. Grid cells in the mEC are periodically activated during locomotion. There is evidence that the theta rhythm, produced by the medial septum with a frequency modulated by locomotor speed, may be crucial to obtain grid cell spatial periodicity (Brandon et al., 2011; Koenig et al., 2011; Hinman et al., 2016; but see Yartsev et al., 2011; Pastoll et al., 2013). Interestingly this theta oscillation appears organized as a traveling wave in the hippocampus, such that there are many theta inputs from the medial septum which just differ by their phases (Patel et al., 2012; Zhang and Jacobs, 2015). In terms of how a traveling theta wave may result in a grid structure, models suggest that the distributed phases of theta oscillatory input to different grid cells gate which ones respond to other shared periodic inputs, since these can only activate grid cells in a specific phase range. Because these inputs would slightly differ from theta in frequency, the grid cell subset which is in the appropriate phase for activation by the shared input cyclically changes to give successive firing of different grid cells and grid cell periodicity (Hasselmo and Shay, 2014; Shay et al., 2016). This idea of conjunction of inputs could relate to a two-layer model of the locomotor CPG, where periodic recruitment of a synergy sometimes depends on the conjunction of inputs from within a PF layer, with a discrete input from a separate TW layer (Saltiel et al., 2016).

A different suggested role for the theta rhythm in the mEC is to give rise to nested gamma oscillations, which appear regulated tightly enough in time, to be described as clock-like (Pastoll et al., 2013). Thus, although the time scales are different, an analogy could be suggested between the locomotor traveling wave and the temporal grid we respectively found in the frog and the cat, and the theta traveling wave and nested gamma clock-like oscillations in the mEC. In locomotion, the temporal grid of critical points subdivides the cycle in different segments; and critical points allow adjustments of these segments when afferent inputs indicate for example that the actual limb position is ahead or behind that expected from CPG activity. Some of these adjustments can be seen as advancing or delaying the CPG program to a later or earlier segment (Figure 4). In the hippocampus, the gamma cycles nested within the theta cycle may also help to represent the environment in segments (Gupta et al., 2012; Pfeiffer and Foster, 2015). And a change in the gamma frequency (slow vs. fast gamma) may promote a change in focus of the segmentation during the theta cycle, from looking ahead/prediction to encoding of actual location or experience, or to looking behind, when navigating or during episodic memory (Gupta et al., 2012; Bieri et al., 2014; Zheng et al., 2016).

In summary, research on the hippocampus/mEC system appears to give an interesting perspective to our results on the locomotor CPG, and suggests some features of generality. Tentatively, this may include a traveling wave giving rise to a grid structure; and using such a grid to allow flexible, yet discrete, adjustments in the segmentation.

## Relationship to Arthur Winfree’s Work on Phase Singularities

In his book, Winfree (1987) first considered a single oscillating cell (squid giant axon, or the sinoatrial node), that was space-clamped, i.e., only time was a consideration. Testing the effect of a brief depolarizing or hyperpolarizing stimulus applied at different times in the inter-spike interval, there is a critical phase and stimulus magnitude where the timing of the post-stimulus spike is no longer predictable. These phase singularities were found both theoretically, e.g., using Hodgkin-Huxley equations, and experimentally. They occurred at a similar time in the squid axon and sinoatrial cycles, at ~38% and ~88% (half-a-cycle apart) of the inter-spike cycle, for the depolarizing and hyperpolarizing stimuli respectively (his Figures 4.3 and 4.1; Best, 1979). The first way that we can relate our work to these results is that we also identified critical points for the effect of phasic protractions and retractions on the Triceps ENG fictive locomotor cycle in the cat (time 0 being the onset of Triceps), and that these occurred at ~38% and ~89% of the cycle, respectively (Figures 1A, 2A). Thus it appears that a very basic result found in a single firing neuron is conserved at the level of an entire CPG.

Winfree (1983) next had the idea that he could easily extend his results to include the consideration of space as well as time. Oscillators were positioned as neighbors inside a rectangular space. By using a traveling wave of activation moving from right to left, a timing gradient was set up along the horizontal dimension of the rectangle. Thus in the space between two consecutive moving vertical wavefronts located some distance apart inside the rectangle, the array of oscillators covered the entire phase spectrum from 0 to 1 along the x-axis of the rectangle. Then applying a stimulus at different positions along the horizontal axis of the rectangle was equivalent to applying stimuli at different phases in time in the previous time-only paradigm. For a depolarizing stimulus, Winfree’s simulations predicted a pair of phase singularities located at the two possible intersections inside the rectangle between a vertical line at the critical phase for that stimulus, and a circle of critical stimulus magnitude from a centrally-applied radially-decaying stimulus applied at that phase (Winfree, 1987, his Figures 6.1 and 6.2). Similar predictions were made for a hyperpolarizing stimulus (his Figures 7.13 and 7.14). These predictions were confirmed in experiments done on the beating heart (Shibata et al., 1988; Winfree, 1989), and also relate to results in oscillating chemical solutions (Zaikin and Zhabotinsky, 1970; Winfree, 1972). Thus the second way that we can relate our work to Winfree results, is that he combined in the same analysis, a phase gradient created by a traveling wave, such as we found in the frog, and critical points at specific phases, such as we found in the cat, to understand where phase singularities will arise in space.

**Figure 9:**
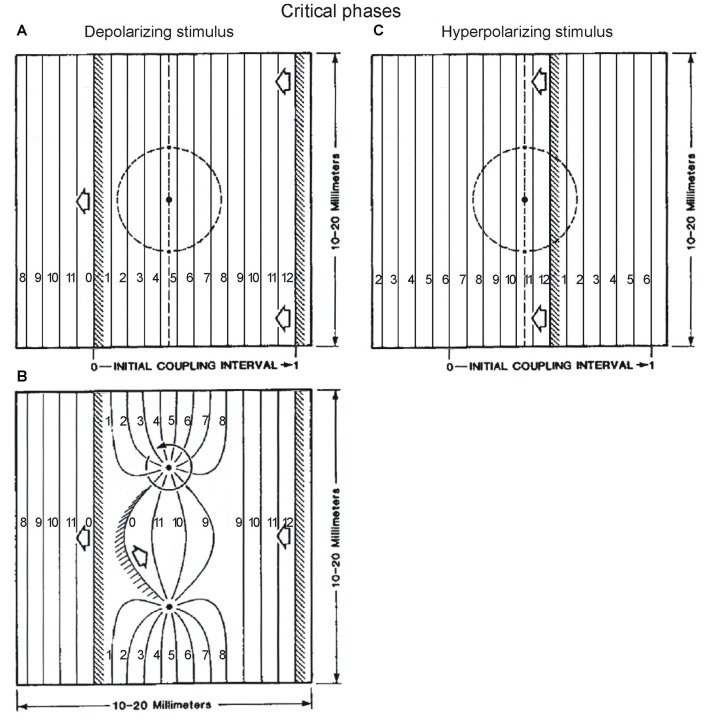
The effect of stimulus delivered at critical phases, examined in space, according to Winfree’s work. A traveling wave from right to left establishes a phase gradient in the rectangular space of tissue. The wavefronts (shaded regions) delimit in space, a cycle divided in equal intervals, as indicated by the isochrons labeled from 0 to 12. The isochron number indicates how long ago the wavefront has passed that location in space. The location of critical phases in the cycle is known from experiments on the effect of depolarizing and hyperpolarizing stimuli given at different times in a space-clamped situation (e.g., firing squid axon). These critical phases are at ~38% and ~88% of the cycle according to Winfree, 1987 chapter 4 (his Figures 4.1–4.5, and 4.9), which is also what we found for the effect of phasic protractions and retractions on the ipsilateral TriLo cycle. In the rectangular space, these correspond to the locations between isochrons 4–5, and isochrons 10–11, as indicated by vertical dashed lines in **(A,C)** respectively. Note that in order to respect these critical phase locations, the wavefronts in **(A,B)** have been moved 3 isochrons earlier in their trajectory, compared to Winfree’s original figures (Figures 6.1 and 6.2 in his 1987 book, chapter 6); and the wavefront in **(C)** has been moved 2 isochrons earlier in its trajectory, compared to the original figure (Winfree, 1987, Figure 7.13). According to Winfree’s work, after delivery of a radially-decaying stimulus (applied at black dot inside dashed circle in **A** or **C**), a pair of rotors is established at the intersection between the critical phase (vertical dashed line), and critical stimulus (dashed circle), as shown in **(B)** for a depolarizing stimulus. In the region of space between the 2 rotors, the isochrons have changed from 4–5 to 10–11, while elsewhere they are much less or not modified. This work from Winfree suggests a potentially interesting interaction between our two experimental results of critical points, and a traveling wave in locomotion. Tentatively, the phase gradient set up rostrocaudally in the spinal cord by a traveling wave, means that the critical points, defined in the time domain, translate into critical locations in the space domain. At these critical locations, afferent inputs could set up phase singularities (rotors), with seemingly consequences on the organization of time in the different spinal cord regions encoding different synergies. Adapted with permission from Winfree (1987), Figures 6.1, 6.2, 7.13.

In Winfree’s simulations and related experiments, the phase singularities are the sites of rotors (pivots of a rotating wave) which organize timing in space. For example, Figures 9A,B (adapted from Winfree, 1987, Figures 6.1 and 6.2) are snapshots of the isochrons (curves connecting sites where phase is the same) immediately before and after a depolarizing stimulus at the critical phase. The cycle between the two wavefronts is divided in 12 intervals, and isochrons indicate regions of space that are synchronous in time. Wavefront isochrons are labeled 0 = 12, and isochrons labeled 1, 2, … indicate that the wavefront was there 1, 2, … units of time previously. The rotors have arisen from a stimulus given in Figure 9A, between isochrons 4 and 5 on the x-axis, at ~38% of the cycle between the wavefronts at the left and right, i.e., at the critical phase for a depolarizing stimulus. We notice that between the two rotors, the isochrons that were 4–5 before the stimulus (Figure 9A), are now 10–11 after the stimulus (Figure 9B), while the other regions do not show much change in their isochrons after the stimulus. If we imagine Figure 9B to be the spinal cord rostrocaudally oriented from right to left, with interneurons located in the space between the two rotors encoding a particular synergy, these simulations suggest a way for a stimulus to change the timing of that synergy. This would occur without affecting the timing of other components of the spinal cord output, encoded by other regions where little or no change occurs in isochrons after the stimulus. This kind of outcome is generally similar to the results in the cat where the timing of only some burst components is modified by afferent proprioceptive inputs. And it relates to the concept of two-layer models of CPG, where one layer maintains a temporal structure, while burst components encoded by another layer can be shifted in time, yet with the overall temporal structure remaining intact.

Finally, it is interesting that the critical phase for a hyperpolarizing stimulus at ~88% of the cycle is between isochrons 10–11 (Figure 9C). Perhaps the change as a result of a depolarizing stimulus in Figures 9A,B, from isochrons 4–5 (critical phase for a depolarizing stimulus) to 10–11 (critical phase for a hyperpolarizing stimulus) is an example of shifts between critical points. Winfree alludes to that observation in the legend of Figure 7.14 of his 1987 book. It remains unclear however how to explain the larger set of critical points that we found in the cat.

In summary, while it has long been suggested to view the CPG as coupled oscillators, it seems that useful insights might specifically come from theoretical considerations as in the ground-breaking work of Arthur Winfree, who emphasized phase singularities as a key to understand the organization of timing in space. This approach starts with the same two ingredients as our experimental evidence in locomotion for: (1) critical points, which we found precisely at the same times as reported by Winfree; and (2) a rostrocaudally traveling wave that would create a phase gradient in space. These two elements are then combined to localize phase singularities in the physical space of the spinal cord, with implications for altering the timing of output components such as synergies, while preserving the overall temporal structure of the cycle. Compared to other systems studied in this way (myocardium, chemical oscillations), the spinal cord and locomotion have the added features that the oscillators encode different synergies, with a specific topography in the spinal cord space. We could thus envision that it might be possible to characterize what the critical loci indicated by the vertical dashed lines in Figures 9A,C may be encoding. And expressing the new timing of the critical locus after the perturbation in terms of where the wavefront would have to be located in the unperturbed cord to result in that timing, might allow comparison to the critical point shifts observed when superimposing the perturbed and control locomotor cycles in the cat (Figure 4).

## Future Directions

Given that the frog research has shed some light on cat locomotion, it would appear useful to further investigate in the frog the spinal cord circuitry that appears to underlie the locomotor synergy sequence, using our suggested two-layer model (Saltiel et al., 2016) to guide experiments. There are now several available techniques for retrograde tracing that can be limited to monosynaptic spread (Wickersham et al., 2007), or that can be made selective by using the Cre-loxP system. A good point of entry would be the synergy A (key muscle = Gastrocnemius) circuitry, since it is at the intersection of the PF layer and TW layer in our model.

For example, the vesicular stomatitis virus (VSV) pseudotyped with the rabies virus envelope, and encoding a fluorescent protein, can be used for retrograde tracing in a wide variety of species (Mundell et al., 2015). When the VSV-G (glycoprotein) gene is deleted from the virus (VSV-∆G), and the rabies virus G gene (RV-G) is supplied separately as trans, one expects a purely monosynaptic retrograde labeling (Beier et al., 2013). Thus their combined injections in GA muscle should selectively label synergy A premotor interneurons. Repeating an application of the RV-G gene at a spinal cord site evoking synergy A should again complement VSV-∆G, this time in synergy A premotor interneurons, resulting in labeling of their monosynaptic inputs. According to our model, this might include for example synergy G premotor interneurons in the PF layer, and interneurons from the TW layer. Coupling this experiment with a different fluorescent protein to label synergy G premotor interneurons could help in distinguishing between these two inputs, if indeed they correspond to different populations.

Another possible method for retrograde tracing is to use the wheat germ agglutinin (WGA) gene in an adeno-associated virus (AAV) vector to carry the Cre gene from GA muscle to synergy A premotor neurons. WGA labeling of premotor neurons from muscle injections has been shown (Harrison et al., 1986; Minic et al., 2016), and WGA tracing has been used in the frog (Levy et al., 2015). Then applying at a spinal cord site evoking synergy A, a Cre-dependent virus with a double-floxed fused WGA-fluorescent protein (Sugita and Shiba, 2005) or TTC (tetanus toxin fragment C)-fluorescent protein (Maskos et al., 2002) should retrogradely label inputs to synergy A premotor neurons.

Indeed, using anatomical techniques, evidence has been found for connectivity between spinal premotor neurons projecting to different motoneurons (Levine et al., 2014), but this has not yet been related to specific synergies. An example with functional implications is the demonstration of unidirectional connectivity between brainstem premotor neurons responsible for successive phases (pharyngeal and esophageal) of swallowing (Broussard et al., 1998). This would represent connectivity within a PF layer. More recently, evidence for different regional distributions of putative RG and PF interneurons has been reported (Griener et al., 2013).

It would also be interesting to search for more evidence for a traveling wave by using a method based on the CaMPARI genetically encoded calcium indicator that would be delivered widely to the spinal cord, using a virus selectively recovered for widespread transduction ability (Deverman et al., 2016). In this method, active neurons when exposed to a 405 nm light permanently photoconvert from a green to a red color. Shining the light at specific phases of the locomotor cycle would allow to see which neurons are active in that phase; and whether there is a rostrocaudal traveling wave for progressively later phases. Progress is being made in getting the light into greater depth up to 1 mm (Flytzanis et al., 2015).

In conclusion, the results in frog locomotion suggest specific ways in which the CPG circuitry could be anatomically investigated. At the same time, the dual perspective from the results in cat and frog locomotion suggests that theoretical approaches, inspired from other fields and disciplines, might be worthwhile to elucidate how critical points, and a traveling wave may be combined into a CPG architecture. Further experimental data such as the anatomical investigations above, may also provide other clues helpful to the theoretical approach.

## Author Contributions

All authors listed have made substantial, direct and intellectual contribution to the work. PS wrote the manuscript, and MCT, KW, AA and EB revised the manuscript.

## Conflict of Interest Statement

The authors declare that the research was conducted in the absence of any commercial or financial relationships that could be construed as a potential conflict of interest.
